# Membranous Nephropathy With Crescents in a Patient With Hashimoto’s Thyroiditis: A Case Report

**DOI:** 10.1097/MD.0000000000000063

**Published:** 2014-08-04

**Authors:** Bijin Thajudeen, Santhosh G. John, Nduka-Obi Ossai, Irbaz B. Riaz, Erika Bracamonte, Amy N. Sussman

**Affiliations:** Department of Nephrology (BT, N-OO, ANS); Department of Medicine (SGJ, IBR); Department of Pathology (EB), University of Arizona Medical Center, Tucson, Arizona, USA.

## Abstract

Membranous nephropathy is a common cause of nephrotic syndrome in adults. It usually occurs secondary to underlying disease processes such as autoimmune disorders, malignancy, infection, and drugs. The presentation of nephrotic syndrome with concomitant precipitous decline in renal function warrants investigation of a coexistent disorder.

We report the case of a 30-year-old male who presented with symptoms and signs of hypothyroidism.

A diagnosis of Hashimoto’s thyroiditis was contemplated based on the presence of high serum levels of antithyroglobulin and antithyroid peroxidase antibodies. Upon initiation of treatment with levothyroxine, patient symptomatology improved; however, the laboratory studies demonstrated continued elevated creatinine, hematuria, and proteinuria, which had not been addressed. Two months following treatment initiation, he had progressive deterioration in renal function and proteinuria. A renal biopsy revealed coexistent necrotizing and crescentic glomerulonephritis and membranous nephropathy.

The final diagnosis was necrotizing, crescentic glomerulonephritis with superimposed membranous nephropathy likely secondary to Hashimoto’s thyrodiitis.

Induction treatment with oral cyclophosphamide and prednisone was started.

At the end of 6 months of treatment, there was improvement in renal function and proteinuria and maintenance treatment with azathioprine and low-dose prednisone was initiated. This case highlights the importance of precise and detailed evaluation of patients with autoimmune diseases such as Hashimoto’s thyroiditis particularly in the presence of active urine sediment. Proper evaluation and diagnosis of such patients has implications on the prognosis and response to treatment.

## INTRODUCTION

Membranous nephropathy is a common cause of nephrotic syndrome in Caucasian adults.^1^ It can be primary or secondary to autoimmune disorders, malignancy, chronic infection, or drugs.^1^Deterioration of renal function in patients with membranous nephropathy can be because of renal vein thrombosis, malignant hypertension, or an associated necrotizing and crescentic glomerulonephritis (NCGN).^2^ NCGN is rare and typically seen in the presence of an underlying disease such as lupus nephritis or because of a distinct, separate disease process such as antiglomerular basement membrane antibodies or antineutrophil cytoplasmic antibody-related (ANCA) pauci-immune glomerulonephritis.^1^ Here we describe a case of membranous nephropathy with P-ANCA-associated NCGN secondary to Hashimoto’s thyroiditis.

## CASE PRESENTATION

A 30-year-old previously healthy Hispanic man presented to the hospital with 2–3 months history of fatigue, somnolence, cold intolerance, arthralgia, dryness of skin, constipation, weight gain, and night sweats. Past medical history was significant for history of a tick bite on the right lower extremity with subsequent development of fever and rash that resolved. He denied smoking and use of alcohol or drugs. Family history was significant only for hypertension.

On physical examination, his vitals were stable with temperature 98.0°F, blood pressure 113/79 mmHg, pulse rate 88/min, and respiratory rate 18/min. There was no pallor, icterus, or edema. Neurological examination revealed delayed ankle jerk and mild cognitive impairment. The remainder of the examination was unremarkable. The laboratory tests are shown as Table 1. Serum creatinine kinase was elevated at 3200 IU/L. Urine analysis demonstrated myoglobin <1 mg/L (0–1 mg/L), specific gravity 1.026, pH 6, protein 100, red blood cells (RBCs) 43 per high-power field, and white blood cells (WBCs) 5 per high-power field. Quantification of proteinuria was not performed at the time. A renal ultrasound was unremarkable. A diagnosis of severe hypothyroidism secondary to Hashimoto’s thyroiditis was established. He was started on levothyroxine and intravenous fluids. His clinical symptoms greatly improved and his creatinine decreased to 1.5 mg/dL, 48 hours after admission to the hospital. The etiology of renal failure was presumed to be due to hypothyroidism and rhabdomyolysis given improvement in serum creatinine with volume expansion. The etiology of the microscopic hematuria and proteinuria evidenced on urine analysis remained uncertain. Two months later, his thyroid stimulating hormone (TSH) decreased to less than 10 μIU/mL with normalization of total T4 and free T4; however, creatinine simultaneously increased to 3.1 mg/dL. Hence, he was referred to the Nephrology Department for further evaluation and management.

**TABLE 1:**
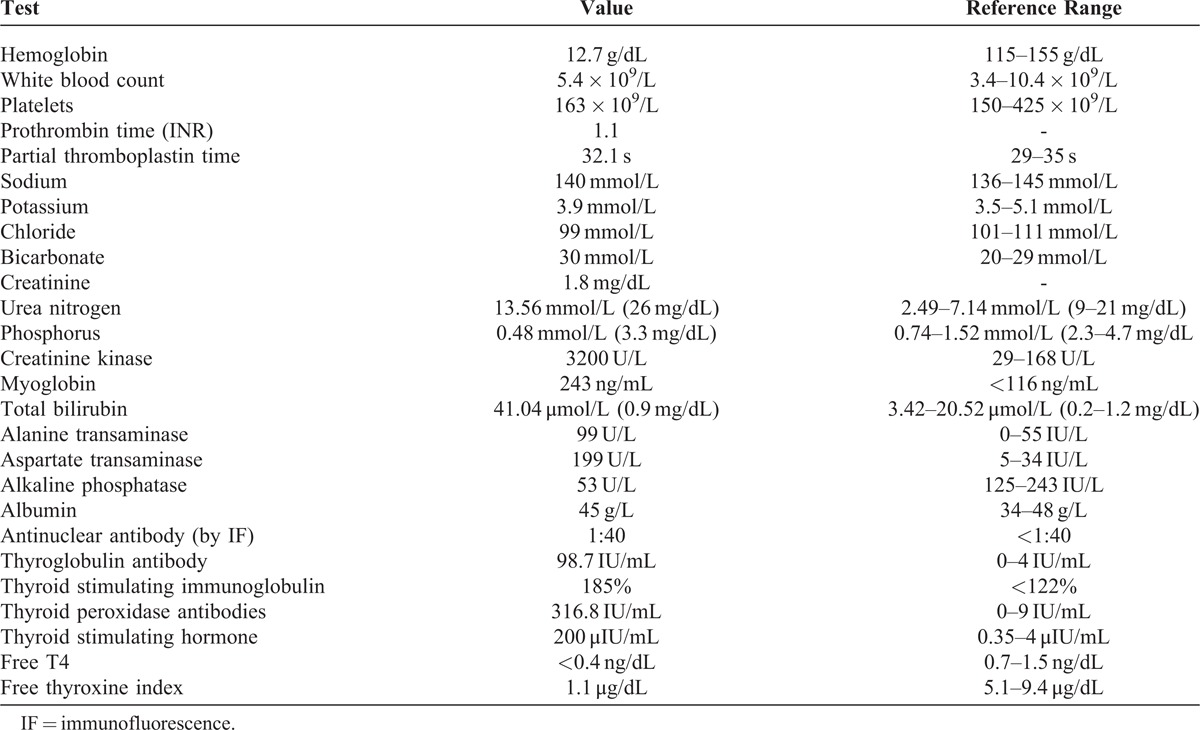
Laboratory Results

**FIGURE 1:**
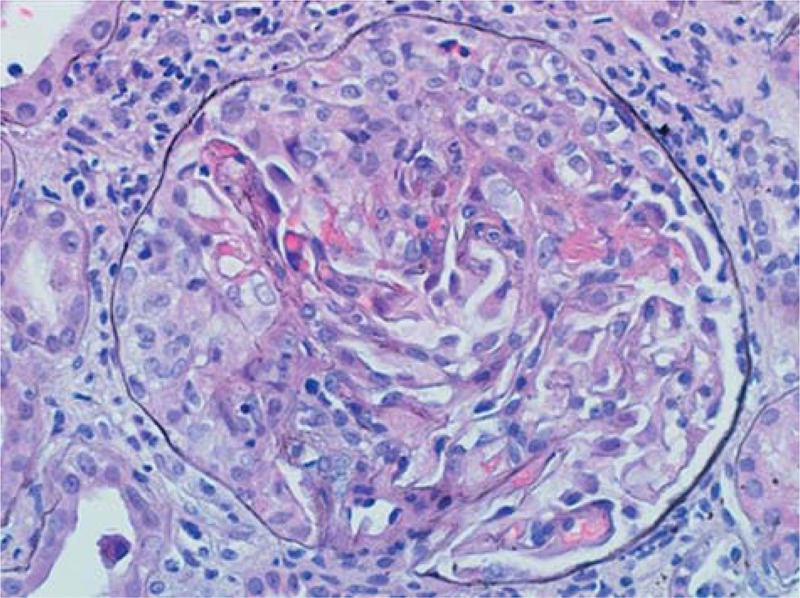
Light microscopy of glomerulus showing normal mesangial areas, slightly thickened capillary loops without inflammation, endocapillary proliferation or obvious immune deposits, and crescent in Bowman’s space (H&E 400×).

**FIGURE 2:**
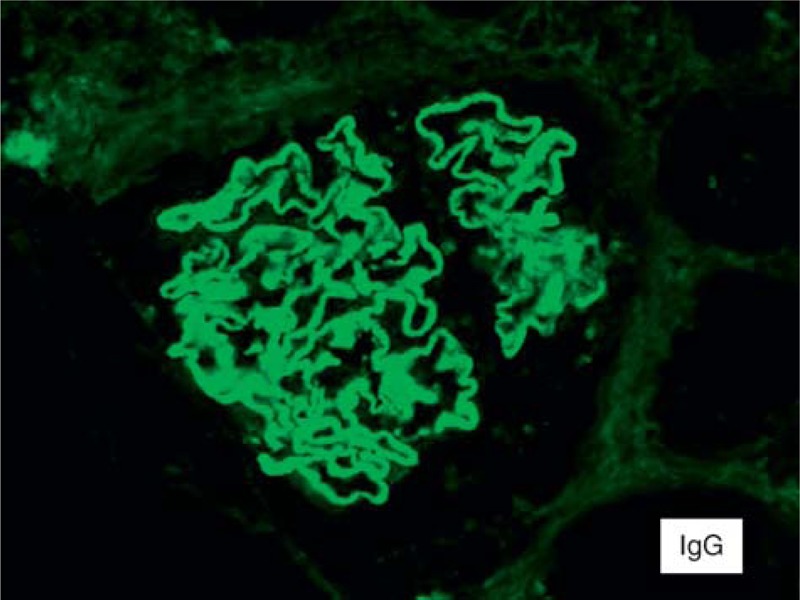
Immunofluorescence microscopy showing fine granular staining for immunoglobulin G (3+) (200×).

**FIGURE 3:**
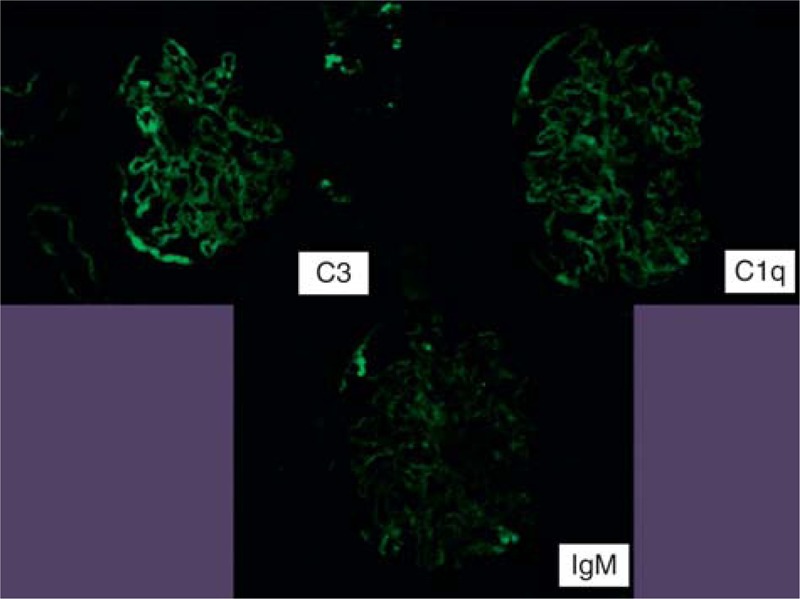
Immunofluorescence microscopy showing staining for immunoglobulin M (trace), C3 (2+), C1q (1–2+) (100×).

A urine analysis demonstrated specific gravity of 1.012, pH 6.5, protein 300, RBC 200/high-power field, WBC 0–1/high-power field. Evaluation of spun urine sediment showed numerous RBCs of which at least 75% had dysmorphic features and occasional RBC casts. A spot urinary protein-to-creatinine ratio was 5.66 g/g creatinine and albumin-to-creatinine ratio was 4.24 g/g creatinine (urine albumin 437.3 mg/dL, urine protein 584 mg/dL, urine creatinine 103 mg/dL). Additional serological workup revealed complement C3 111 mg/dL (0.82–1.85 g/L) and complement C4 26 mg/dL (0.15–0.53 g/L). P-ANCA was positive (by immunofluorescence) and myeloperoxidase (MPO) immunoglobulin G (IgG) antibody was 46 AU/mL (0–19 AU/mL). Serine protease (PR3) IgG antibody was 9 AU/mL (0–19 AU/mL). Antinuclear antibody (ANA), anti-dsDNA, hepatitis panel, HIV, anti-glomerular basement membrane (GBM) antibody, and C-ANCA were negative. Serum electrophoresis and urine electrophoresis revealed no monoclonal bands. Subsequently, arenal biopsy was done (Figures 1–3) that showed glomeruli with normal mesangial areas and slightly thickened capillary loops without inflammation, endocapillary proliferation, or obvious immune deposits. Two-thirds of the glomeruli showed crescents that varied from cellular to fibrocellular. There was presence of moderate patchy interstitial inflammation and moderate-to-severe interstitial fibrosis. Arteries and arterioles appeared normal without evidence of vasculitis. Immunofluorescence microscopy showed fine granular staining for IgG (3+), IgM (trace), C3 (2+), C1q (1–2+), kappa (2+), and lambda (2+). IgG subclass staining could not be done. There was focal segmental staining of glomeruli for fibrinogen (2+) in areas of necrosis. An electron microscopy was not done because of insufficient specimen.

A diagnosis of membranous nephropathy associated with ANCA-mediated crescentic glomerulonephritis likely secondary to Hashimoto’s thyroiditis was made. A negative antiphospholipase A2 antibody lessened the possibility of primary membranous nephropathy. Although there was evidence of significant chronicity, the available literature and relative youth of the patient tended toward the decision to start induction therapy with oral cyclophosphamide and prednisone. The patient tolerated the treatment without any side effects. At the end of 6 months of therapy, his creatinine was 1.5 mg/dL and proteinuria was 1.2 g/g. He was switched to oral azathioprine and small-dose prednisone for maintenance treatment. The plan is to continue the maintenance treatment for a total of 18 months. Repeat MPO IgG antibody was 19 AU/mL and both thyroperoxidase and antithyroglobulin antibodies were within normal limits. The patient tolerated the medications without any side effects.

## DISCUSSION

Hypothyroidism is a known cause of renal failure through multifactorial pathophysiology.^3^ The initial rise in serum creatinine in this case was thought to be because of hypothyroidism (and rhabdomyolysis). Hypofunctioning of the thyroid leads to hypoperfusion of the kidneys from reduced cardiac output and subsequent intrarenal vasoconstriction.^3^ Glomerular filtration rate can decrease by up to 40% as a result of this phenomenon.^4^ This reduction in renal function is usually reversible with treatment of hypothyroidism.^3^ Although the initial rise in serum creatinine in this case was thought to be because of hypothyroidism (and rhabdomyolysis), there was a progressive deterioration of renal function eventually. Given the patient’s history of proteinuria and hematuria on the initial urinalysis, it is possible that there was undiagnosed membranous nephropathy (with crescents) that was proved subsequently on renal biopsy.

Membranous nephropathy associated with Hashimoto’s thyroiditis is well described in literature, but membranous nephropathy associated with crescents and MPO antibodies in the presence of Hashimoto’s thyroiditis is very rare. There are few cases reported in the literature.^5^ The underlying mechanism for glomerular lesions in Hashimoto’s thyroiditis is thought to be due to deposition of thyroglobulin–antithyroglobulin immune complexes in the glomerulus.^6^ During thyroiditis, there is a release of thyroglobulin that results in the generation of antithyroglobulin antibody.^7^In addition, the glycoprotein megalin expressed on thyroid cells in a TSH-dependent manner may also play a crucial role in the immunopathogenesis of glomerular injury in membranous nephropathy.^8^ Other glomerular diseases associated with Hashimoto’s thyroiditis include minimal change disease, IgA nephropathy, membranoproliferative glomerulonephritis, focal segmental glomerulosclerosis, and amyloidosis.^4^

The high titers of MPO antibody (MPO-ANCA) might have played an important role in the pathogenesis of crescentic glomerulonephritis in this case. Patients with a history of thyroid disease are more likely to have MPO-ANCA than PR3-ANCA.^9^ Crescents in the presence of membranous nephropathy may also suggest another underlying disease process, such as lupus nephritis or anti-GBM disease.^2^ Although C1q deposits were found in the glomeruli on immunofluorescence, lupus was ruled out in this case in view of negative anti-dsDNA and normal complements. Moreover, a typical lupus nephritis lesion (except in the case of pure membranous lupus nephritis) would also have associated endocapillary proliferation and concomitant subendothelial deposits, reticular aggregates, and full house staining by immunofluorescence (IgA was lacking in this case). A negative anti-GBM test and the absence of liner staining on immunofluorescence lessened the possibility of anti-GBM disease to the point of exclusion.

The coexistence of autoimmune thyroid disease and ANCA-associated disease has been described in experimental studies.^9^ In these studies, genetic association and cross reactivity between antigens have also been hypothesized as potential mechanisms for this association. A functional polymorphism in the protein tyrosine phosphatase gene, the PTPN22 620 W allele, has been recognized as a predisposing factor for Hashimoto’s thyroid diseases and ANCA positivity.^10^ *PTPN22* is a gene located on chromosome 1p13.3–13.1.^10^ It encodes an 807-amino acid residue protein (also referred to as LYP) that interacts with the tyrosine kinase Csk in the intracellular signaling cascade following T-cell activation.^10^ The autoimmunity-predisposing allele of *PTPN22* is a missense variation that results in a gain of enzymatic function that increases the threshold for T-cell receptor (TCR) signaling.^10^ This impaired amplification of TCR signaling is similar to the predisposing factor for the development of autoimmunity.^10^ Cross-reactivity between thyroid peroxidase (TPO), the enzyme responsible for the synthesis of thyroid hormone and MPO, might also be predisposing to such an association.^11^ TPO belongs to the family of mammalian peroxidases that include MPO, eosinophil peroxidase, lactoperoxidase, and salivary peroxidase.^11^ A homologous relation between the extracellular portions of these molecules might be playing a role in this association.^11^ Both disease processes also have relation to autoimmune diseases such as rheumatoid arthritis, lupus, temporal arteritis, Sjögren’s syndrome, and scleroderma.^9^

Whether there is an association between tick bite and Hashimoto’s thyroiditis in this case is also a matter of debate. Environmental factors such as occupational exposures and infections might play a role in the autoimmune phenotype of genetically susceptible patients. Exposures to infectious agents such as *Staphylococcus aureus* and *Yersinia enterocolitica*/retroviruses have been implicated in the pathogenesis of ANCA vasculitis and autoimmune thyroid disease, respectively.^9^ Similarly, there have been reports of Hashimoto’s thyroiditis and ANCA positivity associated with Lyme’s disease exists in the literature.

Optimal treatment of this condition is not well defined at this point. There is no randomized study looking at the ideal regimen for treatment. Based on the case series reported in the literature, we decided to use oral cyclophosphamide and prednisone in this case. Both intravenous and oral cyclophosphamide has been used for treatment with varying degrees of response.^12,13^ There has also been reports of the use of plasmapheresis (if associated with anti-GBM) and rituximab either as first line or in those not responding to cyclophosphamide.^12,13^ The overall prognosis has been poor despite the use of immunosuppressive therapy.^12,13^ There have been reports of recurrence of membranous nephropathy with crescents after renal transplantation.^14^ Whether a similar recurrence can occur in this case is unknown.

## CONCLUSION

Membranous nephropathy with crescents secondary to Hashimoto’s thyroiditis and positive MPO represents dual glomerulopathy from 2 separate autoimmune processes due to a single underlying disorder. The diagnosis of MGN with ANCA-associated crescentic glomerulonephritis should be considered in patients who present with rapidly progressive glomerulonephritis and nephrotic syndrome. This patient cohort has a poor prognosis, and therefore, prompt recognition is essential for the effective treatment.

## References

[1] Nasr SH Said SM Valeri AM Membranous glomerulonephritis with ANCA-associated necrotizing and crescentic glomerulonephritis. Clin J Am Soc Nephrol. 2009;4:299–308. 19158367 10.2215/CJN.04060808 PMC2637583

[2] Basford AW Lewis J Dwyer JP Membranous nephropathy with crescents. J Am Soc Nephrol. 2011;22:1804–1808. 21903992 10.1681/ASN.2010090923

[3] Iglesias P Díez JJ Thyroid dysfunction and kidney disease. Eur J Endocrinol. 2009;160:503–515. 19095779 10.1530/EJE-08-0837

[4] Koçak G Huddam B Azak A Coexistent findings of renal glomerular disease with Hashimoto’s thyroiditis. Clin Endocrinol (Oxf). 2012;76:759–762. 22106873 10.1111/j.1365-2265.2011.04302.x

[5] Kageyama Y Hamaguchi. K Myeloperoxidase anti-neutrophil cytoplasmic antibody (MPO-ANCA)-positive microscopic polyarteritis (MPA) associated with Hashimoto’s thyroiditis and increased serum rheumatoid factor. Clin Exp Nephrol. 2000;4:335–340.

[6] O’Regan S Fong JS Kaplan BS Thyroid antigen-antibody nephritis. Clin Immunol Immunopathol. 1976;6:341–346. 135663 10.1016/0090-1229(76)90087-8

[7] Ploth DW Fitz A Schnetzler D Thyroglobulin-anti-thyroglobulin immune complex glomerulonephritis complicating radioiodine therapy. Clin Immunol Immunopathol. 1978;9:327–334. 342161 10.1016/0090-1229(78)90104-6

[8] Illies F Wingen AM Bald M Autoimmune thyroiditis in association with membranous nephropathy. J Pediatr Endocrinol Metab. 2004;17:99–104. 14960028 10.1515/jpem.2004.17.1.99

[9] Lionaki S Hogan SL Falk RJ Association between thyroid disease and its treatment with ANCA small-vessel vasculitis: a case-control study. Nephrol Dial Transplant. 2007;22:3508–3515. 17686815 10.1093/ndt/gfm493

[10] Martorana D Maritati F Malerba G PTPN22 R620W polymorphism in the ANCA-associated vasculitides. Rheumatology (Oxford). 2012;51:805–812. 22237046 10.1093/rheumatology/ker446

[11] Hobby P Gardas A Radomski R Identification of an immunodominant region recognized by human autoantibodies in a three-dimensional model of thyroid peroxidase. Endocrinology. 2000;141:2018–2026. 10830285 10.1210/endo.141.6.7506

[12] Tse WY Howie AJ Adu D Association of vasculitic glomerulonephritis with membranous nephropathy: a report of 10 cases. Nephrol Dial Transplant. 1997;12:1017–1027. 9175062 10.1093/ndt/12.5.1017

[13] Barrett CM Troxell ML Larsen CP Houghton DC Membranous glomerulonephritis with crescents. Int Urol Nephrol. 2014;46:963–971. 24217802 10.1007/s11255-013-0593-x

[14] Hill GS Robertson J Grossman R An unusual variant of membranous nephropathy with abundant crescent formation and recurrence in the transplanted kidney. Clin Nephrol. 1978;10:114–120. 359208

